# Immunotherapy strategies targeting tumor-associated macrophages and their mechanisms of action in tumor progression

**DOI:** 10.3389/fimmu.2025.1680455

**Published:** 2025-09-17

**Authors:** Haixia Zhu, Jingjing Shao, Lili Shao, Liuhuan Cai, Chunyan Gu, Qin Ge, Jibin Liu

**Affiliations:** ^1^ Cancer Research Center Nantong, Affiliated Tumor Hospital of Nantong University & Nantong Tumor Hospital, Nantong, China; ^2^ Department of Oncology, Affiliated Tumor Hospital of Nantong University & Nantong Tumor Hospital, Nantong, China; ^3^ Department of Hematologic Lymphoma, Affiliated Tumor Hospital of Nantong University & Nantong Tumor Hospital, Nantong, China; ^4^ Department of Pathology, Nantong Third People’s Hospital & Affiliated Nantong Hospital 3 of Nantong University, Nantong, Jiangsu, China; ^5^ Department of Radiation Oncology, Nantong Tumor Hospital, Affiliated Tumor Hospital of Nantong University, Nantong, China; ^6^ Institute of Oncology, Affiliated Tumor Hospital of Nantong University & Nantong Tumor Hospital, Nantong, China

**Keywords:** tumor microenvironment, tumor-associated macrophages, tumor immunotherapy, immune cell, tumor treatment

## Abstract

The tumor microenvironment significantly influences the aggressive invasive characteristics of human solid tumors, with the infiltration of immune cells being a defining feature of tumor advancement. Among the diverse cell types present in the tumor microenvironment, tumor-associated macrophages (TAMs) stand out as crucial regulatory centers in the interplay between tumors and the immune system. Recent developments in single-cell sequencing technologies, combined with an expanding body of research, have revealed the functional diversity and heterogeneity of TAMs, as well as the mechanisms through which they interact within the tumor microenvironment. This indicates that TAMs could represent innovative targets for therapies aimed at tumors, thus promoting the creation of tailored anti-cancer strategies. This article provides a review of the various types of TAMs, their influence on tumor development and progression, their regulatory functions in tumor activities, and the progress in tumor immunotherapy that focuses on targeting TAMs.

## 1 Introduction

Cancer, as a major disease that significantly threatens human life and health worldwide, has seen its incidence and mortality rates continuously rise in recent years, presenting a severe challenge to the global public health system (1, 2). The tumor microenvironment (TME) is a highly complex and dynamically evolving ecosystem composed of tumor cells, fibroblasts (such as cancer-associated fibroblasts), tumor-associated macrophages (TAMs), and various immune cells, including dendritic cells, T cells, natural killer cells, and myeloid-derived suppressor cells (MDSCs) (3–5). Additionally, it encompasses non-cellular components such as the extracellular matrix, vascular networks, secreted factors, metabolites, oxygen tension, and pH levels. These components exhibit significant spatial and temporal heterogeneity and gradients through intricate cell-cell and cell-matrix interactions. The TME not only directly influences the proliferation, invasion, and metastasis of tumor cells via mechanical scaffolding, nutritional and metabolic supply, and vascular structures, but also regulates the biological behavior of tumors through continuous signaling networks, resulting in diverse phenotypes and fates at various stages (5–10). Macrophages present in the tumor microenvironment are referred to as TAMs. In recent years, an increasing number of studies have demonstrated that TAMs play a significant role in tumor immune regulation and other aspects (11–14). TAMs are macrophages that infiltrate the surrounding tumor tissue; they differentiate from peripheral monocytes and, under the influence of the tumor microenvironment, secrete various cytokines to regulate tumor initiation, metastasis, and invasion (15–17). TAMs are classified into M1 and M2 types, where M1 macrophages exert anti-tumor effects, while M2 macrophages promote tumor progression (18). In tumor tissues, a significant proportion of TAMs differentiate into M2 macrophages, thereby facilitating tumor initiation, invasion, and metastasis. The ratio of M1 to M2 macrophages in TAMs correlates with tumor prognosis, with a higher differentiation of M2-type TAMs being associated with unfavorable outcomes. This indicates that macrophage differentiation has important implications for clinical tumor treatment (19, 20). As research advances, the scientific community’s focus has gradually shifted towards the roles of TAMs in tumor immune evasion, progression, and metastasis, as well as their potential as therapeutic targets (21–23). Therefore, it is essential to investigate the subtypes and functional characteristics of TAMs, elucidate the interaction mechanisms between TAMs and the tumor microenvironment, and conduct research on related anti-tumor strategies and drug development. This will contribute to the formulation of new clinical strategies for tumor treatment and enhance patient prognosis.

## 2 The phenotype of TAMs

**Figure 1:**
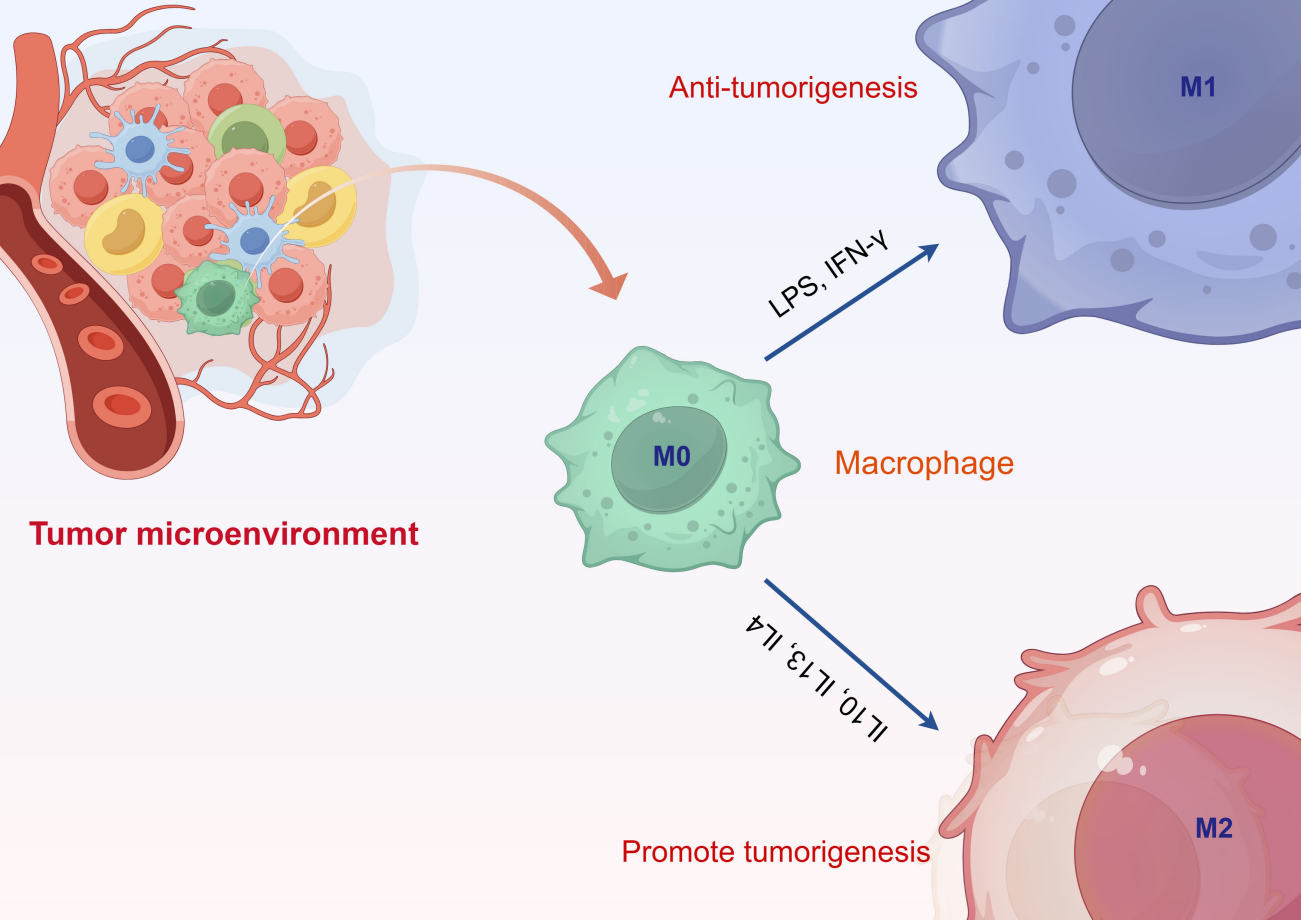
Classification of tumor-associated macrophages.

The classification of TAMs is relatively complex and is dynamically regulated throughout the tumor progression process. They can be broadly categorized into two polarized types: M1 classically activated macrophages and M2 alternatively activated macrophages (**Figure 1**). The microenvironment of tumors significantly impacts the differentiation of macrophages, involving a range of regulatory elements. In this setting, the differentiation of macrophages is influenced by numerous cytokines, metabolites, and interactions between cells. For example, the cathelicidin-related antimicrobial peptide (CRAMP) generated from prostate cancer is capable of attracting immature myeloid progenitors (IMPs) to the tumor microenvironment through chemotaxis, which facilitates their differentiation and polarization into pro-tumor M2-type macrophages (24). In particular, CRAMP enhances the transformation of IMPs into M2-type macrophages by activating p65, thereby modulating the expression of M-CSF and MCP-1, and subsequently triggering the STAT3 signaling cascade. Moreover, the miR-BART11 encoded by the Epstein-Barr virus can impede the differentiation of monocytes into macrophages by targeting FOXP1, thus impacting the secretion of inflammatory cytokines in the tumor microenvironment and fostering the advancement and progression of nasopharyngeal carcinoma and gastric cancer (25). In this context, miR-BART11 directly interacts with the 3’-untranslated region of the FOXP1 gene, inhibiting its expression and consequently influencing the differentiation and functionality of macrophages. Additionally, metabolites within the tumor microenvironment, such as lactate, play a significant role in regulating macrophage differentiation. Lactate interacts with c-Jun, impacting the Notch signaling pathway, which adjusts myeloid cell differentiation and ultimately affects tumor progression (26). These findings illustrate that various factors within the tumor microenvironment interconnect, working together to modulate macrophage differentiation and influence the progression of tumor development. These two phenotypes display significant differences in metabolic pathways, signaling pathways, and cytokine induction (27). M1-type TAMs possess anti-tumor effects, recognizing and killing tumor cells through direct cytotoxic mechanisms and antibody-dependent cellular cytotoxicity. The direct cytotoxic mechanism involves M1-type TAMs identifying tumor cells and releasing tumor-killing factors such as nitric oxide (NO) and reactive oxygen species (ROS) (28). Conversely, M2-type TAMs promote tumor growth. On one hand, tumor cells secrete cytokines such as IL-10, IL-6, and IL-17 to induce the differentiation of macrophages into the M2 phenotype. On the other hand, M2-type TAMs secrete cytokines like IL-4, TGF-β, and M-CSF, facilitating tumor cell growth and participating in tumor infiltration, metastasis, and angiogenesis (29). Additionally, M2-type macrophages can inhibit the activity of T lymphocytes, thereby suppressing the tumor-killing effects of these lymphocytes (30). The cell types of M1 and M2 macrophages are not fixed; during tumor progression, the tumor microenvironment can regulate the mutual conversion between these two types of macrophages, indicating a high degree of plasticity in their cell types.

## 3 The function of TAMs

### 3.1 The immunoregulatory functions of TAMs

TAMs are essential in regulating the immune response, playing a significant role in both tumor immune evasion and immune surveillance. In the tumor microenvironment, these macrophages can dampen immune responses through various mechanisms, facilitating the escape of tumor cells from immune detection. For example, TAMs produce the immunosuppressive enzyme IL4I1, which not only restricts T cell proliferation but also encourages the development of FoxP3+ regulatory T cells (Tregs), thus hindering anti-tumor immune activities (31). Research has indicated that IL4I1 leads to a depletion of phenylalanine, obstructs the mTORC1 signaling pathway, and enhances Treg levels, which in turn compromises the functionality of effector T cells. On the other hand, the polarization state of TAMs affects their immune regulatory roles. Medications like Tasquinimod have the potential to induce a transformation in TAMs from a pro-angiogenic and immunosuppressive M2-like phenotype to a pro-inflammatory M1-like phenotype, subsequently boosting anti-tumor immune responses (32). In the MC38-C215 colon cancer model, administering Tasquinimod resulted in a decrease in tumor-infiltrating CD206+ M2 macrophages, alongside an upsurge in M1 macrophages expressing MHC II and CD86. This alteration in phenotype corresponded with heightened intratumoral production of IL12 and diminished tumor neovascularization. Furthermore, tumor necrosis factor-related apoptosis-inducing ligand (TRAIL) has the ability to reprogram TAMs into an M1-like phenotype, triggering cytotoxic effects on tumor cells and bolstering their immunomodulatory capabilities (33). These findings indicate that the immunomodulatory capabilities of TAMs are complex and adaptable. By influencing the polarization states and cytokine secretion of TAMs, it may be feasible to modify the immune equilibrium within the tumor microenvironment, thereby presenting novel targets and strategies for tumor immunotherapy.

### 3.2 The role of TAMs in tumor metastasis

TAMs are essential players in the spread of tumors. They take part in the metastatic cascade at multiple stages. In breast cancer, TAMs secrete similar factors which promote the migration of the tumor cells. The transforming growth factor beta-induced protein (TGFBI) and Tenascin C released by TAMs, for example, aid in the movement of ovarian cancer cells. Studies have shown that patients with high-grade serous ovarian cancer (HGSC) who have high levels of ascitic proteins secreted by TAMs have decreased progression-free survival (34). Moreover, TAMs enhance the process of angiogenesis and lymphangiogenesis which ultimately provides a conducive milieu for metastatic cell. In colorectal cancer, the infiltration of TAMs is known to drive tumor angiogenesis and lymphangiogenesis, allowing the entry of tumor cells into the blood and lymphatic circulation, thus favouring metastatic spread to distant organs (35). In parallel, in gastric cancers, the distribution of TAMs in regional lymph nodes is related to lymph node metastasis and a high density of TAMs was significantly correlated to pathologically positive lymph nodes and pathological TNM staging. This indicates that the lymphatic metastasis process of gastric cancer may involve TAMs (36). TAMs encourage tumor metastasis in different ways such as enhancing tumor cell migration and invasion, and modifying angiogenesis and lymphangiogenesis, these studies find. To inhibit tumor metastasis targeting, the mechanisms by which TAMs contribute to this metastasis may provide a novel strategy.

### 3.3 The role of TAMs in tumor drug resistance

Research has indicated that in drug-resistant tumors, the ratio of macrophages present within the tumor microenvironment is notably increased, suggesting a role for TAMs in the emergence of drug resistance within tumor tissues (37). The anti-angiogenic medication Zaltrap/Aflibercept works by binding to vascular endothelial growth factor (VEGF) and placental growth factor (PLGF), which inhibits angiogenesis in patients with recurring glioblastoma, resulting in anti-tumor effects. Nevertheless, TAMs can promote resistance to Zaltrap/Aflibercept in patients through the release of matrix metalloproteinase 9 (MMP9), and the cytokines produced by macrophages may act as indicators of unfavorable prognosis in these individuals (38). Studies have shown that exosomal circPLK1 derived from TAMs promotes resistance to the EGFR inhibitor osimertinib in non-small cell lung cancer (39). In both *in vitro* and *in vivo* studies, BRD4 has been demonstrated to drive chemotherapy resistance in colorectal cancer by regulating the expression of PAI-1 in TAMs (40). Furthermore, TAMs can diminish the therapeutic impact of paclitaxel on breast cancer by activating MAPK/ERK kinase (MEK). By either removing macrophages from breast cancer tissues or inhibiting MEK, the sensitivity of breast cancer to paclitaxel can be increased (41). In a mouse model of breast cancer undergoing treatment with doxorubicin and cisplatin, tumor cells in the microenvironment can attract CCR2+ macrophages by releasing CCL2. These macrophages lead to increased vascular permeability of tumor cells through the secretion of MMP9, thus aiding in tumor cell metastasis (42). Additionally, TAMs can also promote cisplatin resistance by regulating Pol η-mediated translesion DNA synthesis in ovarian cancer (43).

**Figure 2:**
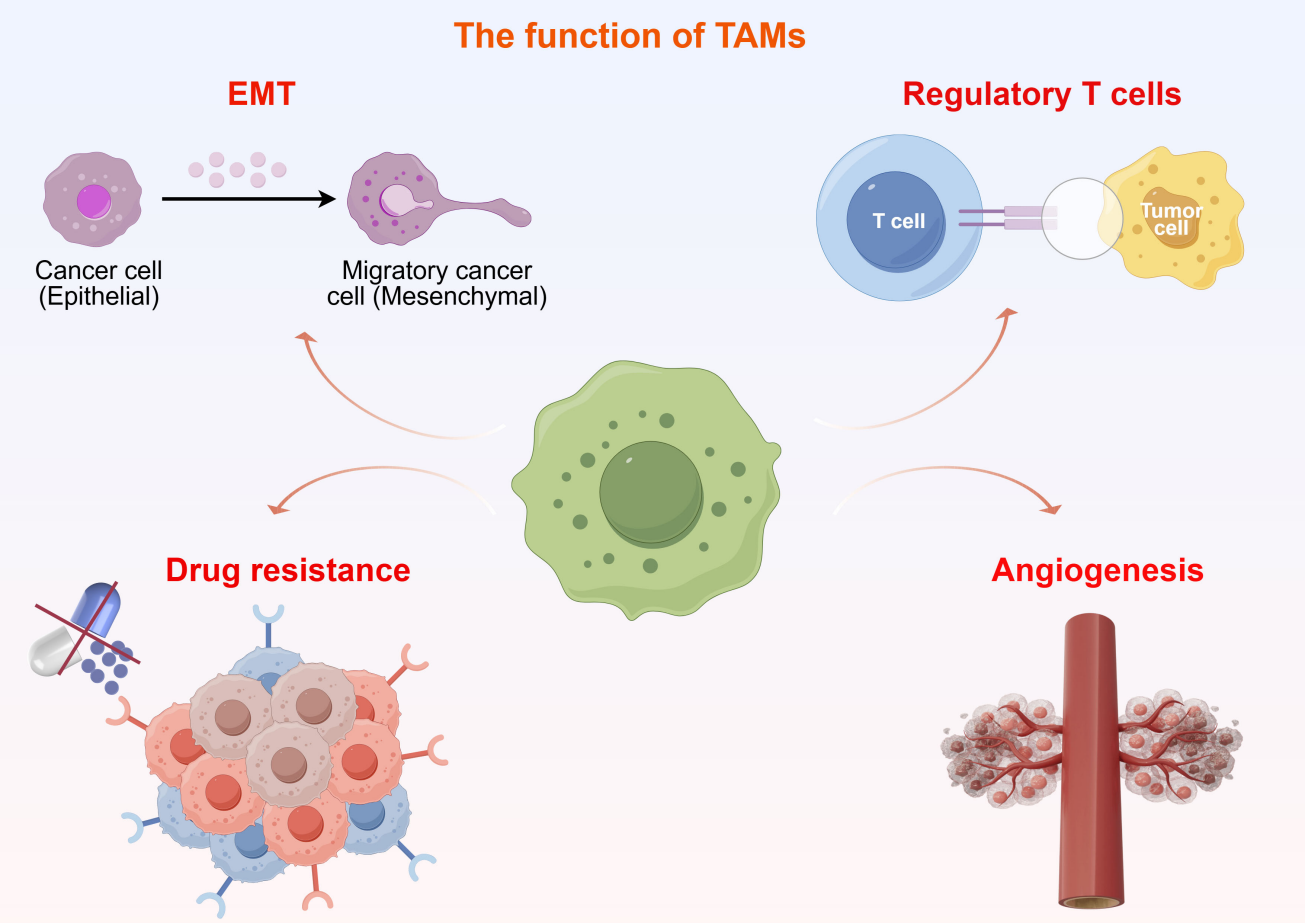
The functions of tumor-associated macrophages.

Overall, the roles of TAMs in immune regulation, metastasis promotion, and drug resistance are notably complex and adaptable. Interventions aimed at modulating their polarization, secretory factors, and associated signaling pathways present promising strategies to enhance the efficacy of immunotherapy and to overcome drug resistance (**Figure 2**).

## 4 The relevant pathways of TAMs in tumors

### 4.1 TGFβ signaling pathway

The TGFβ signaling pathway serves as a pivotal regulatory factor in immunosuppression within the TME, influencing macrophage polarization, tumor cell proliferation, and metastasis (44–46). This pathway modulates macrophage function by promoting their polarization towards an M2-like phenotype, which is associated with pro-tumorigenic activities such as angiogenesis, extracellular matrix remodeling, and immunosuppression (47, 48). Specifically, TGFβ1 induces the aggregation of β4 integrins on the macrophage plasma membrane, enhancing their adhesion to lymphatic endothelial cells (LECs) and driving lymphatic remodeling—a process linked to the metastasis of triple-negative breast cancer (44). Furthermore, TGFβ1 activates RhoA in LECs, leading to lymphatic vessel contraction, increased permeability, and structural alterations, thereby facilitating tumor cell dissemination. The dual role of TGFβ1 in macrophages and LECs emphasizes its significance in fostering a pro-metastatic microenvironment within the TME. In colorectal cancer, TAMs release TGFβ, which facilitates the expression of HIF1α within the cancer cells. As a result, TRIB3 is upregulated, leading to the activation of the β-catenin/Wnt signaling pathway. This sequence of events promotes a stem cell-like phenotype and increases the invasion of tumor cells, further emphasizing the importance of TGFβ in metabolic reprogramming and the advancement of tumors (47). Furthermore, TGFβ-mediated signaling in macrophages has been demonstrated to promote epithelial-mesenchymal transition and the acquisition of cancer stem cell-like properties in hepatocellular carcinoma, further underscoring its role in tumor aggressiveness (49).

### 4.2 WNT signaling pathway

The WNT signaling pathway, well-known for its crucial involvement in embryonic development, maintenance of tissue health, and advancement of cancer, has been identified as a significant modulator of the polarization and function of TAMs. When WNT signaling becomes dysregulated in TAMs, it encourages tumor growth, helps in immune evasion, and supports metastasis, making it an attractive target for therapeutic intervention (50, 51). Recent research reveals that WNT ligands, especially WNT5a, are primarily found in TAMs rather than within cancer cells, highlighting their indirect influence on tumor progression via macrophage-driven processes (52). WNT5a, categorized as a non-canonical WNT ligand, triggers pathways such as CaMKII-ERK, resulting in the release of tumor-promoting substances like CCL2. This, in turn, brings additional monocytes and macrophages into the TME, thereby bolstering tumor progression (44). Additionally, a unique subset of WNT5a+ TAMs has been recognized for its ability to boost tumor invasiveness and support angiogenesis, highlighting the diversity and functional variety of TAMs present within the TME (53). The activation of β-catenin in TAMs has also been demonstrated to drive M2 polarization by regulating key transcription factors such as c-Myc, thereby promoting tumor growth and metastasis (54). In summary, the WNT signaling pathway is a key regulator of TAMs function within TME, influencing their polarization, immunomodulatory roles, and pro-tumorigenic activities.

### 4.3 PI3K signaling pathway

The signaling pathway of PI3K has arisen as a key regulator of TAMs functions, impacting their polarization, metabolic reprogramming, and interactions with various immune and tumor cells (55). The activation of the PI3K pathway within TAMs promotes their transformation into the M2 phenotype. This phenotype is marked by the release of immunosuppressive cytokines, including IL-10 and TGF-β, which serve to diminish the activity of cytotoxic T cells and facilitate immune evasion by tumors (56, 57). The PI3K/AKT signaling cascade is essential for the metabolic adjustment of TAMs within the TME. For example, the PI3K pathway encourages β-oxidation of fatty acids in M2 macrophages, which helps maintain ongoing energy expenditure and bolsters their pro-tumor functions (58). Additionally, activating PI3K promotes the synthesis of eicosanoids, including prostaglandin E2 (PGE2) and leukotrienes, which further aid in tumor progression and immune suppression (48). The relationship between PI3K signaling and lipid metabolism highlights the intricate regulation of TAMs and points to potential therapeutic avenues for interrupting their pro-tumor activities. Recent research indicates that pharmacological blockade of PI3Kγ using agents like TG100–115 can restore the immunosuppressive environment of tumors by lowering immunosuppressive cytokine levels, increasing T cell infiltration, and mitigating immune checkpoint blockade resistance (56, 57). These results imply that targeting the PI3K pathway in TAMs may complement current immunotherapy approaches, offering a promising strategy to enhance clinical outcomes for cancer patients.

### 4.4 TNF signaling pathway

TNFα, an essential pro-inflammatory cytokine, regulates the polarization and activity of macrophages via its receptors, TNFR1 and TNFR2. These receptors initiate unique but interconnected signaling pathways that affect tumor development and immune reactions (44, 59). In melanoma, the CCL20/TNF/VEGFA cytokine secretion phenotype of TAMs has been identified as a negative prognostic factor for cutaneous melanoma (60). Further studies have shown that TAMs induce PD-L1 expression in gastric cancer cells through IL-6 and TNF-α signaling, thereby helping tumor cells evade cytotoxic T cell killing (61). Besides directly affecting tumor cells, TNFα signaling significantly impacts the behavior of TAMs. For example, preadipocytes from the mammary gland that have been treated with TNFα create a pro-inflammatory microenvironment that attracts monocytes and facilitates the migration of epithelial cells; this process is driven by the production of MCP1/CCL2 and mitochondrial reactive oxygen species (62, 63). This cascade of inflammation not only aids in tumor progression but also bolsters the immunosuppressive roles of TAMs, which are essential for evading the immune response and developing resistance to therapies (64, 65). In conclusion, the TNF signaling pathway acts as a key regulator of TAMs function, affecting their polarization, metabolic adjustments, and interactions with cancer cells and various immune elements in the TME. Altering this pathway could potentially hinder the pro-tumor activities of TAMs and improve the effectiveness of cancer treatments.

This section focuses on the critical signaling pathways and their biological significance of TAMs within the tumor microenvironment, specifically addressing the TGFβ, WNT, PI3K, and TNF pathways. In summary, these pathways collectively drive tumor immune suppression, vascular and lymphatic remodeling, epithelial-mesenchymal transition, metastasis, and therapy resistance by regulating TAMs polarization, metabolic and secretory profiles, as well as interactions with tumor and immune cells. This provides important theoretical and clinical insights for the development of multi-target, combined therapeutic strategies.

## 5 Therapeutic strategies for TAMs

### 5.1 Immunotherapy targeting TAMs

In recent years, the targeted immunotherapy of TAMs has emerged as a popular research direction in tumor therapy, which may enhance the body’s anti-tumor immune response by regulating TAMs’ function and phenotype. One strategy utilizes chimeric antigen receptor (CAR) – T cells to kill off specific suppressive TAMs groups. Studies suggest that FR-specific CAR – T cells can specifically remove TAMs that are FR-expressing which have an M2-like immunosuppressive phenotype in syngeneic tumor mouse models (66). Deletion of FRβ+ TAMs leads to a tumor microenvironment enriched in pro-inflammatory monocytes, followed by enhanced influx of endogenous tumor-specific CD8+ T cells which delays tumor progression and prolongs mouse survival. Also, modulating the polarization state of TAMs is an important immunotherapeutic strategy. Small molecule inhibitors of the colony-stimulating factor 1 receptor (CSF1R) can alter the phenotype of the TAMs from M2 (pro-tumor) to M1 (anti-tumor) (67). In a very aggressive 4T1 breast cancer model, administration of supramolecular nanoparticles (DSNs) loaded with dual kinase inhibitors to simultaneously inhibit the CSF1R and mitogen-activated protein kinase (MAPK) signalling, enhances M2 macrophage repolarization to M1 phenotype and the antitumor efficacy. The studies suggest targeting TAMs with immunotherapies has great potential that can be used as a new strategy for cancer.

### 5.2 Drug intervention strategies for TAMs

The aim of pharmacological intervention is to inhibit tumor growth and progression with the modulation of TAMs functions and activities. Medications directly target TAMs and change their phenotype and functions. In cases of chronic lymphocytic leukemia (CLL), blocking the signaling pathway of the colony-stimulating factor 1 receptor (CSF1R) or using clodronate liposomes to kill macrophages can effectively hinder the growth of the leukemia (68). Researchers investigating how to treat leukemia, discovered that depleting macrophages, can induce cellular death in leukemia via the TNF pathway and alter the tumor microenvironment for an anti-tumor effect. Furthermore, some drugs can also alter the interactions between TAMs and tumor cells. The interaction between TAMs and breast cancer cells can be disrupted by either piroxicam or sulindac sulfide in the event of breast cancer (69). According to research, these drugs are capable of reducing RAS expression and downregulating inflammatory/extracellular matrix-related signaling pathways, such as IL-1β, IL-6 COX-2 and PGE2. In addition, they can activate mechanisms of apoptosis and inhibit target factors, such as BCL-2, VEGF-A, MMP-2 and MMP-9, thus inhibiting the evolution of inflammation-driven breast cancer. A range of pharmacological strategies for tumor treatment may alter the functions of TAMs, thereby enhancing the efficacy of cancer therapy.

### 5.3 Regulation of TAMs in combined therapy strategies

Combination therapy boosts the therapeutic efficacy of tumor therapies by using multiple treatment modalities to cooperate against TAMs. The combination of the BRAF inhibitor dabrafenib and the MEK inhibitor trametinib with adoptive cell transfer in melanoma is shown to enhance anti-tumour effect. According to studies, there is increased infiltration of TAMs and Tregs in the tumor microenvironment with dabrafenib monotherapy. However, inclusion of trametinib decreases these immunosuppressive cell populations with an increase in T cell numbers within the tumor, which in turn increases *in vivo* cytotoxicity. In addition, the two treatments together leads to an increased expression of melanocyte antigens and MHC molecules and activation of a broad spectrum of immune response genes. For the control of non-small cell lung cancer (NSCLC), treatment with the new generation erlotinib analogue TD-92 in combination with anti-PD-1 therapy is showing robust anti-cancer responses (70). TD-92 reduces the number of CD11b+F4/80+ TAMs with a subsequent downregulation of CSF-1R without any considerable alteration in other immune cell populations. The immune contexture in the TME will be remodeled to improve anti-tumor immune response, which will lead to better outcomes in NSCLC combination treatment. The combination therapy approaches regulate TAMs through distinct mechanisms, providing innovative methods to improve the efficacy of tumor therapies.

## 6 Future perspectives

TAMs serve as vital immune regulatory entities within the tumor microenvironment, significantly contributing to the initiation, progression, metastasis, and resistance to therapies. While recent advances in research and therapeutic approaches targeting TAMs have achieved notable milestones, there remain various challenges and opportunities for future investigations. To begin with, the diversity and plasticity of TAMs phenotypes and functions call for a more comprehensive understanding of their underlying molecular mechanisms. By utilizing cutting-edge technologies such as single-cell sequencing, spatial omics, and the integration of multi-omics, scientists aim to elucidate the characteristic heterogeneity and dynamic alterations of TAMs in the tumor microenvironment with greater accuracy, facilitating more targeted interventions. Furthermore, the effective modulation of TAMs polarization to encourage their shift from the pro-tumor M2 phenotype to the anti-tumor M1 phenotype continues to be a key focus for future immunotherapeutic strategies.

The emergence of small molecule medications, nanocarrier drug delivery systems, and gene editing tools offers crucial support for this goal. Moreover, approaches leveraging CAR-T cell technology that specifically target and eliminate immunosuppressive TAMs subpopulations exhibit promising potential and are anticipated to improve the efficacy of current anti-tumor immunotherapeutics. The complexity of the tumor immune microenvironment suggests that a singular focus on modulating TAMs alone is unlikely to fundamentally reverse tumor immune suppression. Future combination therapy strategies must explore the intricate interactions between TAMs, other immune cells, tumor cells, and stromal cells. This comprehensive approach aims to establish a multi-dimensional therapeutic network that enhances both the response rate and durability of immunotherapy. Furthermore, clinical translation remains a critical bottleneck that hinders the application of TAMs-related research findings. Future efforts must strengthen the integration of basic research with clinical trials to promote the clinical validation of novel TAMs-targeting drugs and therapeutic regimens. Additionally, identifying suitable populations through biomarkers for personalized treatment is a crucial direction for future research. In summary, future research on TAMs will deeply integrate technological innovations, such as single-cell analysis and multi-omics analysis, with clinical applications. This integration aims to uncover the mechanisms of tumor immune evasion and treatment resistance, promote multidisciplinary collaborative development, and ultimately provide more effective and safer treatment options for cancer patients.
